# Supplementary material for: Acquired Resistance to Decitabine Associated with the Deoxycytidine Kinase A180P Mutation: Implications for the Order of Hypomethylating Agents in Myeloid Malignancies Treatment
Source: Int J Mol Sci. 2025 May 25;26(11):5083. doi: 10.3390/ijms26115083 (PMC12154022; doi:10.3390/ijms26115083)
Supplement: Supplementary file 1 [file ijms-26-05083-s001.zip › ijms-3557477-supplementary.pdf]

International Journal of
*Molecular Sciences*

# Acquired Resistance to Decitabine Associated with the Deoxycytidine Kinase A180P Mutation: Implication for the Order of Hypomethylating Agents in Myeloid Malignancies Treatment

**Kristina Simonicova [1], Lubos Janotka [1,2], Helena Kavcova [1], Ivana Borovska[1], Zdena Sulova [1], Albert Breier [1,3,*], and Lucia Messingerova [1,3,*]**

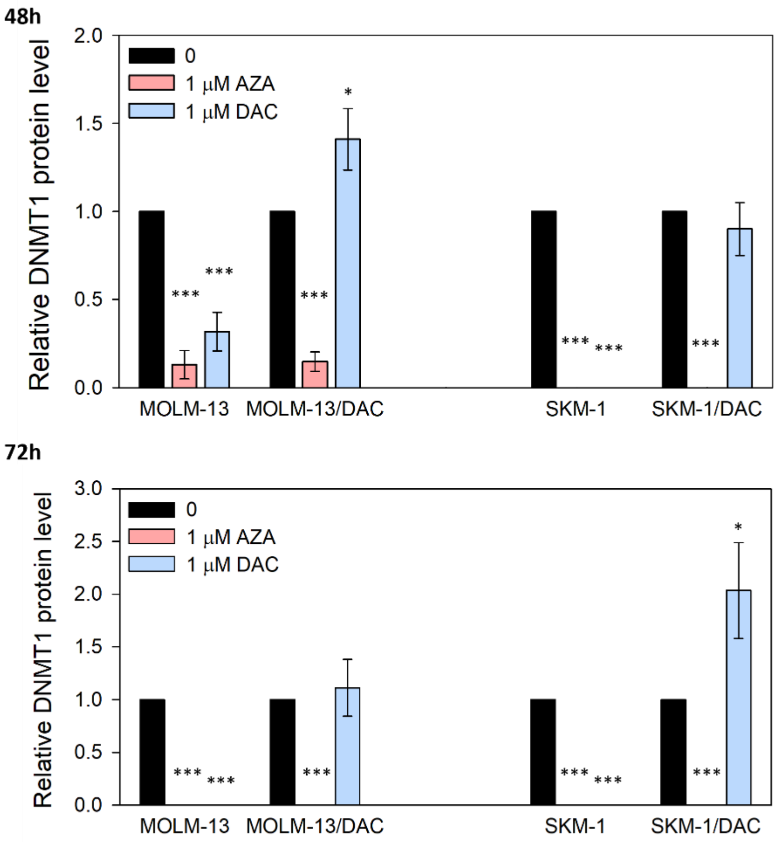


**Figure S1:** The optical densities of the protein bands for DNMT1 (Figure 2A) quantified by densitometry and summarized in the bar plots. Statistical significance is as follows: * $p \leq 0.05$; ** $p \leq 0.01$; *** $p \leq 0.001$.

International Journal of
*Molecular Sciences*

MDPI

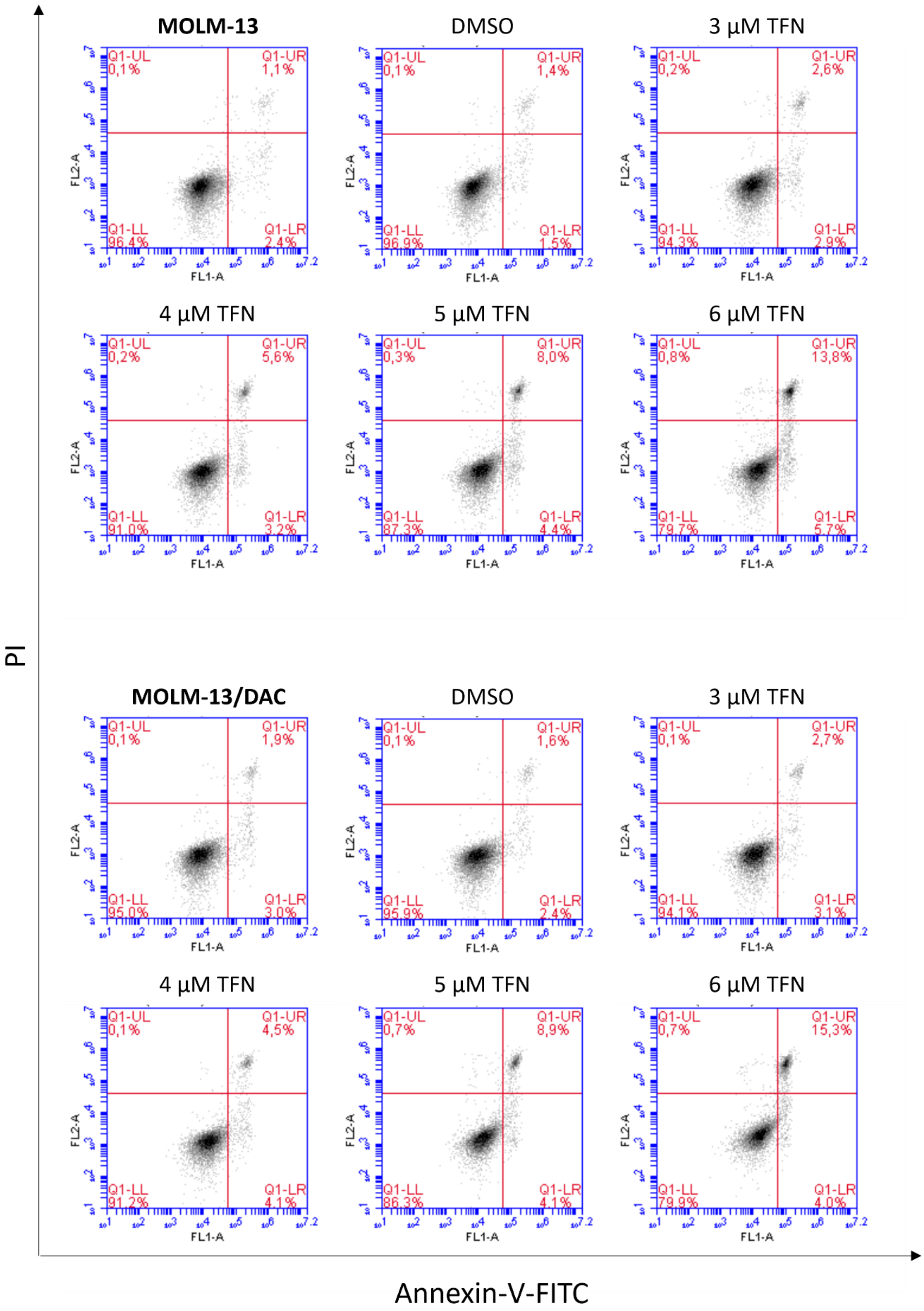


**Figure S2:** Representative dot plots of Annexin-V-FITC and PI assay for MOLM-13 and MOLM-13/DAC treated with TFN for 72 hours, with TFN added every 24 hours (Figure 6C).

International Journal of *Molecular Sciences*

MDPI

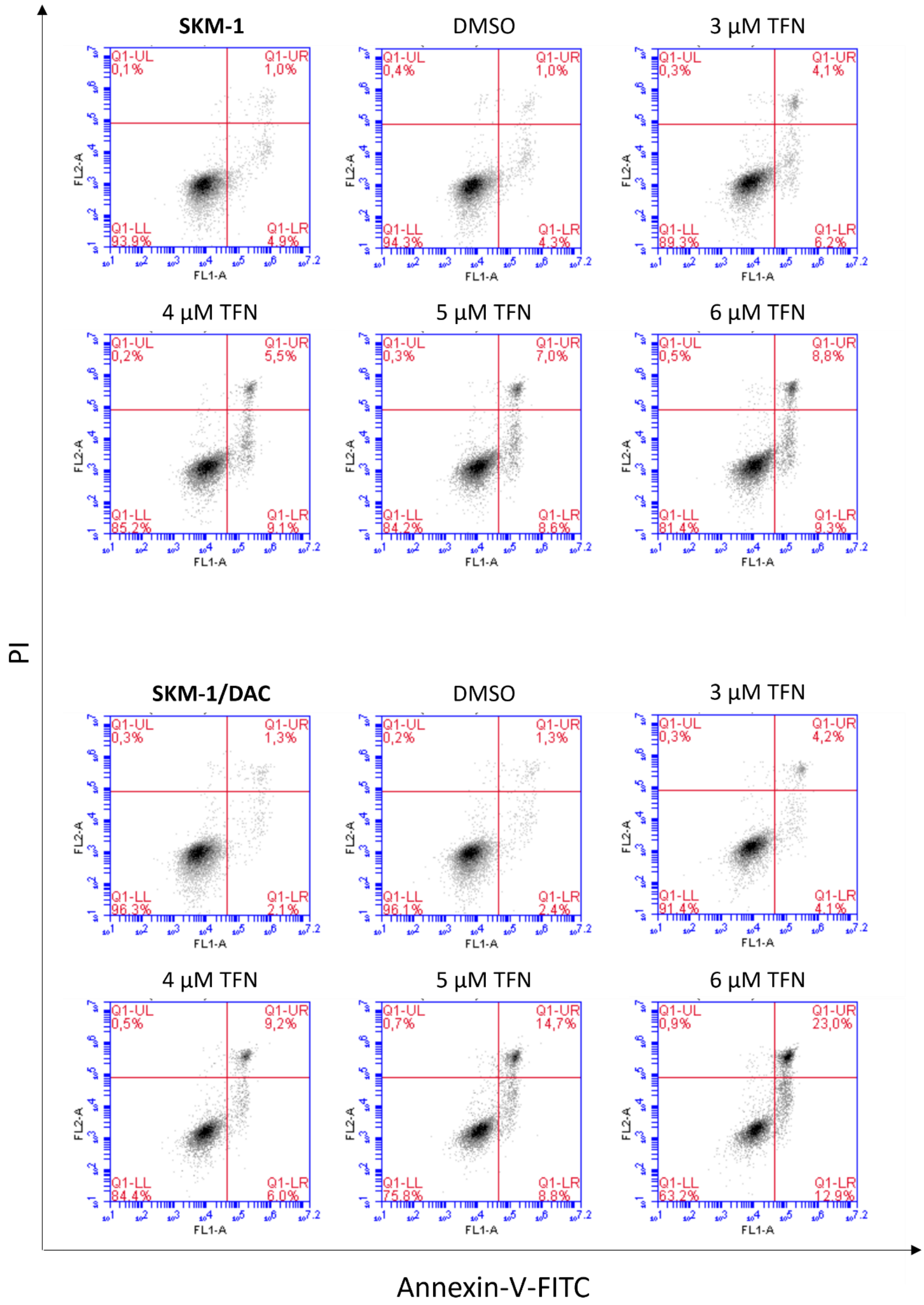


**Figure S3:** Representative dot plots of Annexin-V-FITC and PI assay for SKM-1 and SKM-1/DAC treated with TFN for 72 hours, with TFN added every 24 hours (Figure 6C).
